# Is the MIND diet useful for polycystic ovary syndrome? A case-control study

**DOI:** 10.1186/s12905-024-03090-3

**Published:** 2024-05-09

**Authors:** Mina Darand, Narges Sadeghi, Zahra Salimi, Mahlagha Nikbaf-Shandiz, Asieh Panjeshahin, Hawal Lateef Fateh, Mahdieh Hosseinzadeh

**Affiliations:** 1 Prevention of Cardiovascular Disease Research Center, Shahid Beheshti University of Medical Sciences, Tehran, Iran; 2 Department of Nutrition, School of Public Health, Shahid Sadoughi University of Medical Sciences, Yazd, Iran; 3 Department of Nutrition, School of Allied Medical Science, Ahvaz Jundishapur University of Medical Sciences, Ahvaz, Iran; 4 Nutrition and Metabolic Diseases Research Center, Ahvaz Jundishapur University of Medical Sciences, Ahvaz, Iran; 5 Student Research Committee, Faculty of Nutrition and Food Technology, Shahid Beheshti University of Medical Sciences, Tehran, Iran; 6 Nursing Department, Kalar Technical Institute, Garmian Polytechnic University, Kalar, Kurdistan Region, Iraq; 7 Research Center for Food Hygiene and Safety, School of Public Health, Shahid Sadoughi University of Medical Sciences, Yazd, Iran

**Keywords:** PCOS, Polycystic ovary syndrome, Polycystic ovarian syndrome, MIND diet

## Abstract

**Background:**

Polycystic ovary syndrome (PCOS) is the most prevalent cause of ovulatory infertility and endocrine abnormalities in reproductive-age women. Although the MIND diet has been introduced to improve brain function, evidence shows that the MIND diet is rich in beneficial food groups that can have a preventive effect on other metabolic disorders. The present study was conducted to investigate the association between adherence to the MIND diet and PCOS.

**Methods:**

This age and BMI frequency-matched case-control study was conducted on 216 women between January 2018 and March 2019 in Yazd, Iran. PCOS was diagnosed based on Rotterdam criteria. Participants were selected by convenience sampling method. The validated 178-item food frequency questionnaire was used to assess the usual dietary intake. Logistic regression was used to estimate the association between the MIND diet and PCOS.

**Results:**

The findings of the present study showed a significant inverse association between adherence to the MIND diet and PCOS in the crude model (OR for T3 vs. T1: 0.12 (95% CI: 0.05–0.25), P-value < 0.001) and multivariable-adjusted model including energy intake, age, BMI, waist circumference, marital status, pregnancy history, drug use history, education and physical activity (OR for T3 vs. T1 = 0.08 (95% CI: 0.03–0.19), P-value < 0.001). Adherence to the MIND diet had a protective effect of 92%.

**Conclusion:**

Although the results of the present study showed that higher adherence to the MIND diet is associated with a lower risk of PCOS, more studies are needed to confirm these findings in the future.

## Background

One of the common endocrine metabolic disorders in women of reproductive age is polycystic ovary syndrome (PCOS), which is associated with reproductive, metabolic, and psychological features [1]. The prevalence of PCOS in women of reproductive age is 6 to 18% depending on the diagnostic criteria used and the population studied [2–5]. PCOS was diagnosed based on two of three following Rotterdam criteria: [1] anovulation and/or irregular menstruation (infrequent periods) [2], biochemical and/or clinical hyperandrogenism, and [3] polycystic ovaries (≥ 12 follicles measuring 2–9 mm in diameter and/or an ovarian volume > 10 mL in at least one ovary) [6]. PCOS is associated with intrinsic insulin resistance (IR), which in turn worsens the hormonal and clinical features of polycystic ovarian syndrome [1, 7, 8]. Rates of overweight, obesity and central obesity are higher in women with PCOS than those without PCOS which aggravates IR [9–11]. The first line of treatment considered for PCOS is lifestyle modification including adopting a healthy diet, regular exercise, and psychological support alongside drug therapy [12]. Previous reports suggest that, the diet of patients with PCOS is high in carbohydrates and fat, which increases the lipid inflammatory environment and IR of patients to some extent [13] and causes the progression of the disease. On the other hand, dietary modification and following the DASH, hypocaloric, Mediterranean, low-glycemic, and low-carbohydrate diets had improved BMI, insulin resistance, menstrual irregularity, and decreased testosterone levels in PCOS patients [14].

The MIND diet (a combination of the Mediterranean-DASH diet) has recently been recognized as a new dietary pattern, including 15 components, 10 of which are brain-healthy foods (green leafy vegetables, other vegetables, whole grains, beans, nuts, berries, olive oil, fish, chicken, and wine) and 5 of which are brain unhealthy foods (butter or margarine, cheese, red meat, fast foods or fried foods, and sweets or pastries) [15]. Although the association between the MIND diet and PCOS has not been investigated so far, few studies have examined the effect of this dietary pattern on chronic diseases. For instance, Mohammadpour et al. (2020) reported that adherence to the MIND diet significantly reduced general obesity but did not affect the likelihood of metabolic syndrome and abdominal obesity [16]. In contrast, Aminianfar et al. (2020) did not find a significant relationship between adherence to the MIND diet and the presence of central and general obesity in adult participants [17]. Although we have not yet reached definitive results about the effect of the MIND diet on PCOS, it seems that the components of this diet (especially one of its main components, olive oil) help to improve oxidative stress and, as a result, insulin resistance which is one of the major risk factors associated of PCOS [18–20].

Due to the continuous increase in the prevalence of PCOS and its related risk factors in recent decades and the increase in the rate of obesity, the prevalence of unhealthy dietary patterns and following them, we aimed to examine the association between adherence to the MIND diet and PCOS in the Iranian population.

## Methods

### Study design and participants

**Fig. 1:**
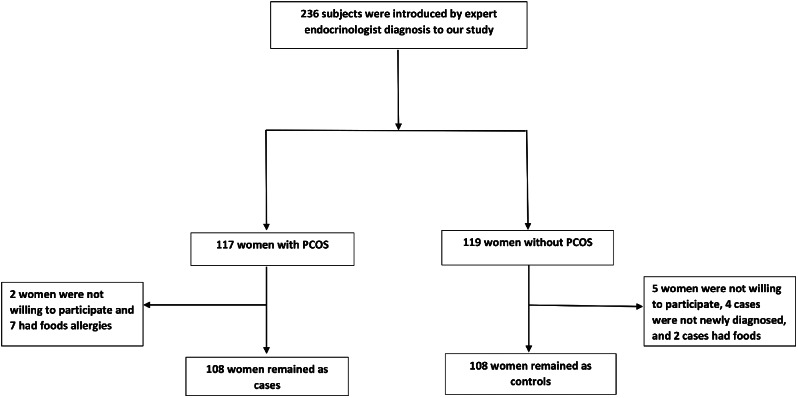
The participants’ recruitment procedures

This frequency-matched case-control study was conducted on 216 women between January 2018 and March 2019. A sample of 108 PCOS newly-diagnosed patients (aged 18 to 45 years) was selected from women referred to Yazd Diabetes Clinic and Khatam Clinic in Yazd. Women with PCOS were diagnosed by an endocrinologist and based on Rotterdam criteria and the presence of at least two of the three following criteria: menstrual irregularities, clinical or biochemical signs of hyperandrogenism, and polycystic ovaries (≥ 12 follicles measuring 2–9 mm in diameter and/or an ovarian volume > 10 mL in at least one ovary) [21–23]. Women without a history of diseases such as hypothyroidism, hyperprolactinemia, congenital adrenal hyperplasia, Cushing syndrome or food allergies, and type 1 diabetes; without a history of using medications such as hormonal drugs, contraceptive pills, or other medicines that could change the androgens levels; women who did not drink alcohol and were not smokers; women who did not follow a specific diet in the last year and did not take nutritional supplements in the past three months and non-pregnant and non-lactating women were recruited. The control group included 108 women without PCOS (lacking Rotterdam diagnostic criteria) who had been referred to other departments of the same clinic such as orthopedics, dentistry, or optometry. Healthy controls were matched to PCOS women based on age and BMI. About 236 subjects were introduced by expert endocrinologist diagnosis to our study (117 subjects for the case group and 119 for the control). Finally, for the case group: 2 women were not willing to participate, and 7 had allergies to foods. for the control group: 5 women were not willing to participate, 4 cases were not newly diagnosed, and 2 had allergies to foods; as a result, 216 women including 108 cases (response rate (92%)) and 108 controls (response rate (90%)) completed study based on matching for age and BMI. Although the number of subjects in the control group was slightly higher than the case group, but after matching for age and BMI with the case group, 108 participants remained in each group. Other inclusion criteria were almost identical for the case and control groups. The participants’ recruitment procedures are represented in Fig. 1.

### Sample size calculation

Due to the limited number of similar articles, the appropriate reference for determining the sample size, considering alpha of 0.05 and a power of 90%, assuming that there is a 20% difference in adherence to dietary pattern in the two groups (P1 = 40%, P2 = 60%), and a 10% probability of sample loss, the minimum required sample size was calculated to be 108 women in each group.

P1 = the ratio of people who followed the dietary pattern among the women without.

PCOS P2 = the ratio of people who followed the dietary pattern among the women with PCOS.

$$n=\frac{{\left({Z}_{1-\alpha /2}\sqrt{2PQ}+{Z}_{1-\beta }\sqrt{{P}_{1}{Q}_{1}+{P}_{2}{Q}_{2}}\right)}^{2}}{{\left({P}_{1}-{Q}_{1}\right)}^{2}}$$

Where $$P=\frac{{P}_{1}+{P}_{2}}{2}$$, $$Q=1-P$$, $${Q}_{1}=1-{P}_{1}$$, $${Q}_{2}=1-{P}_{2}$$

### Anthropometric measurements

Body weight and height (in a fasting condition, with light clothes and no shoes) were measured using Omron digital scale to the nearest 0.1 kg and a nonstretched wall-mounted tape measure to the nearest 0.1 cm, respectively. BMI was computed as the ratio of measured weight in kilograms to height in meters squared and waist circumference (WC) was measured using a non-stretchable tape measure to the nearest 0.5 cm.

### **Physical activity assessment and other covariates**

Physical activity level was evaluated by an International Physical Activity Questionnaire-Short Form (IPAQ-SH) and responses were presented to Metabolic Equivalent Task minutes per week (MET-min/week) [24]. Based on the level of physical activity, people were divided into 3 categories: inactive people, moderate people and active people. Inactive people: reported meth less than 600 MET-minute/ week Moderately active individuals: minimum reported MET greater than 600 MET-minute/ week People with intense meth activity more than 3000 MET-minute/ week [25, 26]. Needed information including age, marital status, pregnancy history, Drug used history (anti-diabetic and anti-hypertensive drugs) and education was obtained using a validated self-administered questionnaire.

### Dietary intake assessment

In a direct interview by a blinded nutritionist, participants’ typical dietary intake over the previous year was obtained using a 178-item semiquantitative food frequency questionnaire (FFQ) [27], which its validity has been approved in the previous studies [28]. The frequency intake of each food item was reported as daily, weekly, monthly, or yearly. Individuals’ food intake was converted to grams using the household scale guideline. Then, total energy and nutrient intake were calculated by transferring food intake (g/d) to Nutritionist IV.

### MIND diet score

The MIND diet score includes 15 dietary components, 10 of which were recognized as brain-healthy food categories (green leafy vegetables, other vegetables, berries, nuts, beans, poultry, fish, whole grains, olive oil, and wine). The remaining items were known as brain-unhealthy food categories (red meats, butter and margarine, cheese, pastries and sweets, and fried/fast foods). In the present study, due to the lack of information, the score of wine consumption was not considered. As a result, 14 food categories were included in the MIND dietary pattern. The participants were categorized into tertile groups based on their intake of 14 components. Those in the lowest, middle, and highest tertiles of brain-healthy food intake were assigned scores of 0, 0.5, and 1, respectively. Conversely, individuals in the lowest tertile of brain-unhealthy food intake received a score of 1, while those in the middle and highest tertiles were assigned scores of 0.5 and 1, respectively. The total Mind score for each participant was calculated by adding up the scores for all dietary items. Finally, each participant was given a score ranging from 0 to 14 based on this calculation [29].

### Statistical analysis

Kolmogorov-Smirnov test was used to examine the normal distribution of quantitative variables and then, categorical and quantitative variables were presented as frequency (percentage) and mean ± standard deviation (SD), respectively. Chi-squared and an independent t-test was performed for inter-group differences of quantitative and qualititative variables, respectively. Also, the comparison of dietary intakes between MIND tertiles was done by the one-way ANOVA test. Multivariate logistic regression was used in different models to investigate the association between MIND diet and PCOS. Model 1 was adjusted for energy intake. Further adjustment was for age, BMI, waist circumference, marital status, pregnancy history, drug use history and education. Physical activity was additionally adjusted in the model III. Data analysis was done using SPSS software version 24 (IBM, Armonk, NY, USA) and a P-value less than 0.05 was considered statistically significant.

## Results

Table 1 represents the general characteristics of participants in both groups (women with and without PCOS). The distribution of age, BMI, physical activity, marital status, pregnancy history, drug use history, and education was not different between the two groups (P˃0.05). The mean waist circumference was marginally significantly higher in women with PCOS than in healthy women. (P˃0.05).

**Table 1:** General characteristics and physical activity of women with and without PCOS

Variables	Case (n = 108)	Control (n = 108)	P value^*^
**Age (y)**	28.95 ± 7.16	30.45 ± 7.17	0.126
**BMI (kg/m2)**	27.10 ± 4.88	26.63 ± 4.87	0.482
**WC (cm)**	82.74 ± 10.77	79.74 ± 11.62	0.051
**Physical activity (n (%))**			0.525
Low	38(35.2)	34(31.5)	
Moderate	33(30.6)	40(37)	
High	37(34.3)	34(31.5)	
**Marital status**			0.778
Single	39(36.1)	41(38)	
Married	69(63.9)	67(62)	
**Pregnancy history**			0.583
No	45(41.7)	49(45.4)	
YES	63(58.3)	59(58.3)	
**Drug use history** ^†^			0.891
No	61(56.5)	62(57.4)	
YES	47(43.5)	46(42.6)	
**Education**			0.275
Illegal	1 (0.9)	0	
Lower than diploma	13 (12)	16(14.8)	
Diploma & Associate’s degree	37(34.3)	26(24.1)	
Bachelor	47(43.5)	59(54.6)	
Master and above	10(9.3)	7(6.5)	
**MIND diet score**	6.10 ± 1.70	7.72 ± 2.17	< 0.001

BMI: body mass index; MET: metabolic equivalent; PCOS: polycystic ovary syndrome; WC: waist circumference. For quantitative variables mean ± SD and for qualitative variables frequency (%) were used.

* Independent t test for quantitative variables and x^2^ test for categorical variables conducted.

^†^ Anti-diabetic and anti-hypertensive drugs

Table 2 reports the characteristics of the study participants across tertiles of MIND diet scores. After MIND diet scores were categorized into three tertiles, no significant differences were observed in mean age, BMI, WC, marital status, pregnancy history, drug use for PCOS, education, and physical activity. Nevertheless, the prevalence of PCOS with increased adherence to the MIND diet significantly decreased (*P* < 0.001).

**Table 2:** Characteristics of the study participants across tertiles of MIND diet scores

Variables	Tertiles of MIND Score			P-value**
	T1 (≤ 5.50)	T2 (6–7.50)	T3 (8≤)	
Age (year)	29.72±7.37	28.55±6.99	30.67±7.13	0.220
**BMI (kg/m2)**	26.72±5.06	26.91±5.15	26.96±4.47	0.949
**WC (cm)**	81.66±11.48	80.57±11.88	81.40±10.67	0.843
**Marital status**				0.935
**Single**	28(36.1)	25(38)	27(38)	
**Married**	69(63.9)	67(62)	67(62)	
**Pregnancy history**				0.935
No	32 (42.7)	29 (55.4)	33 (43.4)	
**Drug use history** ^†^				0.075
No	40 (53.3)	32 (49.2)	51 (67.1)	
YES	35 (46.7)	33 (50.8)	25 (32.9)	
**Education (n (%))**				0.125
Illegal	1 (1.3)	0	0	
Elementary	2 (2.7)	3 (4.6)	7 (9.7)	
High school & Diploma	34(45.3)	26(40)	20(26.3)	
Bachelor	30(40)	33(50.8)	43(56.6)	
Master’s degree and higher	8(10.7)	3(4.6)	6(7.9)	
**Physical activity (n (%))**				0.455
Low	30(40)	17(26.2)	25(32.9)	
Moderate	21(28)	24(36.9)	28(36.8)	
High	24(32)	24(36.9)	23(30.3)	
**PCOS**				0 < 0.001
Yes	54(28)	36(36.9)	18(36.8)	
No	21(28)	29(44.6)	58(76.3)	

BMI: Body Mass Index; WC: Waist Circumference; HC: Hip Circumference; MIND: Mediterranean-DASH Intervention for Neurodegenerative Delay; PCOS: Polycystic ovary syndrome

For quantitative variables mean ± SD and for qualitative variables frequency (%) were used.

^**^For qualitative and quantitative variables Chi-square test and One-way ANOVA were used respectively.

^†^ Anti-diabetic and anti-hypertensive drugs

Dietary intakes of study participants based on tertiles of MIND diet scores are presented in Table 3. Participants in the highest tertile of the MIND diet score had significantly greater intakes of carbohydrates, magnesium, folate, green leafy vegetables, other vegetables, whole grains, fish, and beans than those in the first tertile (*P* < 0.05). Conversely, their intake of fat, cholesterol, saturated fatty acids, monounsaturated fatty acids, butter, margarine, cheese, red meat and products, fast fried foods, and pastries and sweets were significantly lower (*P* < 0.05).

**Table 3:** Dietary intakes of study participants based on tertiles of MIND diet scores

Variables	Tertiles of MIND Score			P-value^**^
	T1 (≤ 5.50)	T2 (6-7.50)	T3 (8 ≤)	
Energy intake (kcal)^*^	2047.77±653.89	2242.46 ±797.51	2039.20±728.14	0.183
Carbohydrate (% of total daily energy)	55.99±0.07	58.27±0.07	62.74±0.05	<0.001
Protein (% of total daily energy)	15.66±0.03	15.79±0.03	16.40±0.02	0.276
Fat (% of total daily energy)	31.28±0.05	29.01±0.05	24.41±0.05	<0.001
Cholesterol (mg/100Kcal)	124.71±48.73	122.82±60.11	104.06±38.10	0.020
SFA (gr/1000Kcal)	11.19±2.84	9.94±2.26	7.80±1.76	<0.001
MUFA (gr/1000Kcal)	10.64±2.61	9.89±2.45	7.79±1.69	<0.001
PUFA (gr/1000Kcal)	11.13±8.41	10.66±5.83	11.77±6.96	0.659
Calcium (mg/1000Kcal)	375.76±129.93	374.90±112.39	395.77±90.98	0.443
Magnesium (mg/1000Kcal)	119.69±18.98	126.43±21.09	136.29±21.10	<0.001
Vitamin B6 (mg/1000Kcal)	1.70±0.73	1.82±0.80	1.83±1.12	0.623
Folate (µg/1000Kcal)	129.77±31.48	138.11±4149	153.66±33.93	0.001
**Food groups**				
Green leafy vegetable (gr/1000Kcal)	8.60±9.88	11.67±9.70	22.22±13.35	<0.001
Other vegetables (gr/1000Kcal)	70.42±50.57	101.14±63.09	135.92±71.50	<0.001
Berries (gr/1000Kcal)	1.83±3.43	1.76±2.78	1.87±3	0.978
Olive oil (gr/1000Kcal)	0.16±0.69	0.66±1.13	0.50±1.78	<0.001
Nuts (gr/1000Kcal)	4.33±5.46	6.43±7.07	4.90±4.91	0.094
Whole grains (gr/1000Kcal)	6.08±9.59	25.75±32.03	53.89±42.90	<0.001
Fish (gr/1000Kcal)	2.97±2.93	4.46±4.29	6.77±4.53	<0.001
Beans (gr/1000Kcal)	13.88±8.96	16.15±13.57	23.71±12.46	<0.001
Poultry (gr/1000Kcal)	15.39±17.50	16.94±19.88	17.86±15.64	0.686
Butter, margarine (gr/1000Kcal)	3.90±5.25	3.15±5.12	0.74±1.82	<0.001
Cheese (gr/1000Kcal)	12.36±11.78	8.88±7.07	8.17±7.39	0.012
Red meat and products (gr/1000Kcal)	34.88±18.80	28.28±22.36	17.39±10.45	<0.001
Fast fried foods (gr/1000Kcal)	19.56±11.57	15.36±12.72	11.97±1.63	0.001
Pastries and sweets (gr/1000Kcal)	23.30±12.90	21.32±22.38	19.91±16.69	0.017

MUFA, Monounsaturated Fatty Acid; PUFA, Ponounsaturated Fatty Acid; SFA, Saturated Fatty Acid; MIND, Mediterranean-DASH Intervention for Neurodegenerative Delay; PCOS, Polycystic ovary syndrome

* Data reported on mean ± standard deviation (SD)

**Obtained from one way Anova

Statistically significant difference (P-value<0.05)

Crude and multivariable-adjusted odds ratios (95% confidence intervals) for PCOS across tertiles of MIND score are demonstrated in Table 4. There was a significant inverse association between adherence to the MIND diet and the odds of PCOS in the crude model. Women in the highest tertile of the MIND diet score compared to those in the lowest tertile had 88% lower odds of PCOS (OR for T3 vs. T1: 0.12; 95% CI: 0.05–0.25, P-trend < 0.001). This association remained significant after adjustment for energy intake, age, BMI, waist circumference, marital status, pregnancy history, drug use history, education, and physical activity (OR for T3 vs. T1: 0.08; 95% CI: 0.03–0.20, *P* < 0.001).

**Table 4:** Odds ratio and 95% confidence interval for occurrence of the PCOS across tertiles of MIND diet score

PCOS	Tertiles of MIND score			P-value^*^	P-trend
	T3 (8 ≤)	T2 (6-7.50)	T1 (≤ 5.50)		
No. of cases	54	36	18		
Crude	1.00	0.48 (0.23-97)	0.12(0.05–0.25)	0.001	< 0.001
Model1	1.00	0.37 (0.17–0.78)	0.09 (0.04–0.20)	< 0.001	< 0.001
Model2	1.00	0.39 (0.18–0.84)	0.09 (0.03–0.20)	< 0.001	< 0.001
Model3	1.00	0.40 (0.18–0.86)	0.08 (0.03–0.20)	< 0.001	< 0.001

MIND: Mediterranean-DASH Intervention for Neurodegenerative Delay; PCOS, Polycystic ovary syndrome

*Third tertile compared to first tertile

Model 1: Adjusted for energy intake

Model 2: additionally, adjusted for age, BMI, waist circumference, marital status, pregnancy history, drug used history and education

Model 3: additionally, adjusted for physical activity

## Discussion

To the best of our knowledge, this is the first case-control study that investigated the association between adherence to the MIND diet and odds of PCOS. A significant inverse association between adherence to the MIND diet and PCOS was found, in such a way that whatever the score adherence to the MIND diet was higher, the occurrence of PCOS was lower.

Although no study examines the association between the MIND diet and PCOS, some studies investigated the relationships between the MIND diet and other metabolic disorders relevant to PCOS, such as obesity, metabolic syndrome, and CVD [30–32]. According to the Mohammadpour et al. study, higher adherence to the MIND diet was associated with an increased risk of general obesity but a reduced risk of low-HDL-C levels. However, adherence to the MIND diet did not affect the risk of metabolic syndrome or abdominal obesity [32]. Aminianfar et al. reported that MIND diet adherence was not significantly correlated with both central and general obesity among adults [31].

Although the causes of PCOS are not yet fully understood, insulin resistance has been implicated as a significant factor [33]. Being overweight and obese can worsen insulin resistance and features of metabolic syndrome, which is also a common finding in PCOS [34, 35]. The MIND dietary pattern is a combination of the Mediterranean (MD) and Dietary Approaches to Stop Hypertension (DASH) dietary patterns. It is believed to have some advantages over these two eating patterns. The advantage of the MIND dietary pattern is assigning separate groups for green leafy vegetables and berries, as well as cakes and pastries [34]. The beneficial effects of the MIND diet may have been relevant to the use of olive oil as a major source of dietary fats and phenolic compounds [36]. Some trial studies reported that oleic acid had anti-inflammatory properties and improved insulin resistance [37]. As it is clear from the results of our study, with the increased adherence to the MIND dietary pattern from tertile 1 to 3, the mean magnesium intake has increased significantly. Components of the MIND diet are rich sources of magnesium, which has a crucial role in regulating several biological processes in the human body [38]. Specifically, magnesium regulates the insulin receptors and improves insulin receptor sensitivity by increasing tyrosine kinase activity [39]. Magnesium also down-regulates the inflammatory response by inhibiting NF-κB, as well as magnesium reduces insulin resistance by restoring antioxidant enzyme activity and scavenging oxygen radicals [40–43].

Previous studies showed a strong association between insulin resistance and elevated serum homocysteine levels in PCOS patients and obese women [44, 45]. On the other hand, there is an association between inadequate folate intake and higher circulating homocysteine concentrations [46]. The MIND diet, as a plant-based diet, is a rich source of folic acid due to its recommendation of whole grains, beans, fruits, and vegetables, especially green leafy vegetables, which appear useful for PCOS patients [47].

Here are some strengths attributed to the current study. This is the first case-control study that investigated the association between adherence to the MIND diet and odds of PCOS. Moreover, enrollment of newly diagnosed subjects declined the recall bias. However, it should be noted that this study had some limitations due to the case-control design, and no causal associations can be identified. Due to the retrospective nature of the FFQ, the probability of recall bias should be addressed. The residual confounders cannot be removed while we have controlled for possible confounding. This study was conducted on a small population of women, so it cannot be generalized to all PCOS patients, and more studies on a larger scale should be done.

## Conclusion

The results of the current study showed a significant inverse association between PCOS and adherence to the MIND diet. Given the limitations we had in the study, further mechanism-based investigations on a larger scale are needed to confirm the results.

## Data Availability

The datasets used and/or analysed during the current study are available from the corresponding author on reasonable request.

## Abbreviations

PCOS: Polycystic ovary syndrome
ESHRE/ASRM: European Society for Human Reproduction and Embryology/American Society for Reproductive Medicine
MIND diet: Mediterranean-DASH Intervention for Neurodegenerative Delay
IPAQ-SH: International Physical Activity Questionnaire-Short
MET: Metabolic Equivalent Task
FFQ: Food Frequency Questionnaire
TLGS: Tehran Lipid and Glucose Study
BMI: Body Mass Index
PA: Physical Activity
WC: Waist Circumference
HC: Hip Circumference
MUFA: Monounsaturated Fatty Acid
PUFA: Polyunsaturated Fatty Acid
SFA: Saturated Fatty Acid
